# On predicting time to completion for the first stage of spontaneous labor at term in multiparous women

**DOI:** 10.1186/s12884-017-1345-1

**Published:** 2017-06-12

**Authors:** Björn Gunnarsson, Eirik Skogvoll, Ingibjörg Hanna Jónsdóttir, Jo Røislien, Alexander Kr Smárason

**Affiliations:** 1 Department of Research, Norwegian Air Ambulance Foundation, Holterveien 24, 1448 Drøbak, Norway; 2 Faculty of Medicine, Norwegian University of Science and Technology, Trondheim, Norway; 3 Department of Obstetrics and Gynecology, Akureyri Hospital, Akureyri, Iceland; 4 Department of Health Studies, University of Stavanger, Stavanger, Norway; 5 Department of Biostatistics, Institute of Basic Medical Sciences, University of Oslo, Oslo, Norway; 6 Institute of Health Science Research, University of Akureyri, Akureyri, Iceland

**Keywords:** Labor progression, Spontaneous labor, Active labor, Partogram, Birthweight, Body mass index, Rupture of the membranes, Robson’s classification

## Abstract

**Background:**

Labor that progresses faster than anticipated may lead to unplanned out-of-hospital births. With the aim to improve planning of transportation to birthing institutions, this study investigated predictors of time to completion for the first stage of labor conditional on cervical opening (conditional time) in multiparous women at term.

**Methods:**

We performed a retrospective analysis of partograms for women in Robson’s group 3 who delivered at one hospital from 2003 to 2013. A generalized additive mixed model was fitted, accounting for possible non-linear relationships between the predictor variables and outcome, e.g. the time from each cervical measurement to full dilation, using multiple measurements for each woman. The following predictors were included: cervical dilation (cm), parity (1, 2, or ≥3 previous vaginal births), oxytocin infusion (no/yes), epidural (no/yes), maternal age (years), maternal height (cm), body mass index (BMI, kg/m^2^), birthweight (kg), spontaneous rupture of membranes (no/yes). A modified regression model with gestational age (days) instead of birthweight was used to predict conditional time to full cervical dilation for combinations of the most relevant predictors.

**Results:**

A total of 1753 partograms were included in the analysis. The strongest predictors were birthweight, epidural and oxytocin use, and spontaneous rupture of membranes, along with cervical measurements. For birthweight, there was an almost 40% increase in time to full cervical dilation for each 1-kg increment. Conditional time was on average 23% longer in cases with epidural use and 53% longer in cases requiring oxytocin augmentation. Spontaneous rupture of the membranes shortened conditional time by 31%. Maternal age was not associated with the outcome, while increasing BMI and parity modestly reduced conditional time.

**Conclusions:**

Higher parity, lower fetal weight (gestational age), and spontaneous rupture of the membranes are associated with more rapid labor.

**Electronic supplementary material:**

The online version of this article (doi:10.1186/s12884-017-1345-1) contains supplementary material, which is available to authorized users.

## Background

Labor patterns vary widely, and both slow and fast labor are associated with complications [1–4]. We have studied unplanned out-of-institution births [5], which might occur because labor progresses faster than anticipated. A majority of the women experiencing such births are multiparous women with singleton, cephalic pregnancies in spontaneous labor at term. Improved prediction of time to completion of dilation in this group could improve planning and management of transportation to birthing institutions.

Many studies have examined the total length of the stages or phases of labor, or the median time of dilation in 1-cm integers (traverse time) [4, 6–8]. However, there is little consensus on the definitions of labor onset or the transition from latent to active phase [9, 10]. The shape of the classic Friedman curve is also contended because the average rate of cervical dilation appears to increase over time without distinct points of acceleration or deceleration [10, 11]. This gradual transition between the latent and active phases of the first stage of labor makes it difficult or impossible to objectively identify the onset of active labor. Average labor curves and tables showing median traverse time in centimeter integers can help diagnose abnormally slow labor progression but are not designed to estimate time to completion of cervical dilation conditional on the cervical opening and other factors known to be associated with the length of labor, including maternal age [12], height [13], body mass index (BMI) [14], parity [8], gestational age [15], and birthweight [6]. As well, timing of admission to the delivery unit [16], epidural analgesia [15, 17], and oxytocin use [18] are known to be associated with length of labor. Clinical protocol with early amniotomy and early oxytocin [19]–but not amniotomy alone [20]–is associated with slightly shorter time from admission to delivery.

The first stage of labor is normally the longest, and the transition to the second stage of pushing and imminent delivery is an important decision point for pre-hospital health workers. Little research, though, has been conducted to improve methods to predict when the transition is likely to happen conditional on cervical opening and other covariates. Therefore, we aimed to use factors associated with the speed of labor to predict time to transition from first to the second stage, using data from multiparous women with singleton, cephalic pregnancy in spontaneous labor at term.

## Methods

### Data material

We analyzed partograms for all women in Robson’s group 3 (multiparous, in spontaneous labor, no previous Cesarean section, gestational age ≥37 weeks, and single, cephalic pregnancy) who delivered at Akureyri Hospital in Iceland from January 1, 2003 to December 31, 2013. This rural hospital handles approximately 430 births annually. Midwives attended all the deliveries and recorded and monitored labor progression by plotting each manual cervical measurement on a partogram, providing a graphical view of labor progression. The frequency of cervical measurements was not standardized, but they were generally taken shortly after arrival at the labor ward and again as clinically indicated. The midwives also recorded information about spontaneous rupture of membranes and oxytocin and epidural use on a pre-printed form. Information about artificial rupture of membranes and descent of the fetal head were not entered consistently and, therefore, could not be used for analysis. Cases of emergency Cesarean sections or void partograms (no cervical measurements) were excluded.

### Primary outcome variable

Women were frequently admitted in advanced labor and did not have a cervical measurement before 4-cm dilation. We, therefore, extracted the time and dilation in centimeters for all cervical examinations of at least 4 cm and calculated the time to full dilation of 10 cm. We refer to this time to full dilation conditioned on cervical dilation as *conditional time*. Onset of pushing was used as a proxy for time to full dilation when this was not confirmed with vaginal examination.

### Statistical analysis

Data for continuous variables were summarized as mean (SD) and range, while data for categorical data were summarized as n, percentage and range. Student’s t-test was used for between-group comparisons of continuous variables, and Pearson’s Chi-square test for categorical data.

Labor progression speeds up non-linearly, so in order to explore possible predictors of conditional time, a model capable of handling non-linearity must be applied. The framework of generalized additive models (GAM), a generalization of the traditional generalized linear model (GLM), allows for non-linear associations between the predictors and the outcome by fitting a smooth spline (i.e., a series of polynomials), rather than a straight line [21]. Additionally, the partograms studied included repeated cervical measures for each birth which were uneven in both number and spacing, so this intra-correlation had to adjusted for. We thus fitted a generalized additive mixed model (GAMM), a generalization of GAM allowing for multiple measurements on each individual, to the data. The outcome variable (conditional time in hours) was heavily skewed, and was therefore log-transformed before statistical analysis to improve normality and model fit. The coefficients were back-transformed (exponentiated) to the original time scale after fitting of the model. The log-transformation and corresponding back transformation results in a model with multiplicative effects of the covariates on the original time scale.

Nine potential explanatory variables were included in the GAMM. The categorical variables were parity (1, 2, or 3 previous vaginal births), epidural use (no/yes), spontaneous rupture of membranes before first cervical examination ≥4 cm (no/yes), and augmentation of labor with oxytocin during the first stage (no/yes). The continuous variables were maternal age (years), height (cm), BMI (kg/m^2^), birthweight (kg), cervical measurements (cm), and length of gestation (days). The use of pushing as a proxy for completion of dilation (*n* = 242, 13.8%) had minimal effects on the outcome (not shown). To calculate BMI, we used the last weight measurement before delivery with a few missing values (*n* = 72, 4.1%), as self-reported pre-pregnancy weight had more missing values (*n* = 242, 13.8%). However, the selection of weight for BMI calculations had minimal effects on the model outcome (not shown).

To select the optimal statistical model, we applied Akaike’s Information Criterion (AIC) [22]. AIC is a weighing between parsimony and model fit to the data, providing an objective measure of the “goodness” of a model. Lastly, we fitted a GAMM with only the covariates that could be determined before hospital arrival and used this model to produce curves and a table of predictions for conditional time. We did so first using the same population and then a given a set of values for the model covariates.

*p*-values <0.05 were considered to be statistically significant. Confidence intervals were calculated using the non-parametric bootstrap [23]. Calculations were performed with R version 3.1 [24].

## Results

**Fig. 1:**
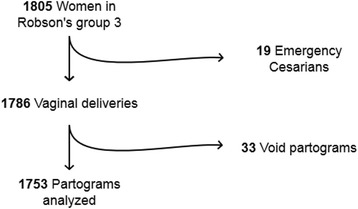
Flow of all women who fulfilled the study criteria

Figure 1 shows the flowchart for the 1805 women who met the study criteria. Cases of emergency Cesarean sections or void partograms due to unplanned out-of-hospital birth (1 unplanned at home, 2 in an ambulance) or delivery immediately upon arrival to the hospital were excluded, leaving 1753 partograms for analysis. The majority of women (*n* = 1734, 98.6%) were Caucasian. Forty (2.3%) women had instrumental deliveries, all of which were vacuum extractions that did not exclude them from further analysis. Women who received oxytocin infusion and/or epidurals were included in the main analysis (*n* = 404, 23.0%). Spinal blocks were not performed during labor in the sample.

The median cervical dilation at admission was 5 cm (range: 0.5–10 cm). Most women (*n* = 1411, 80.5%) measured ≥4 cm, and 966 (55.1%) ≥5 cm. Other variables are summarized in Table 1. Epidural analgesia was used in 312 cases (17.8%), increasing from 14.2% during the first 3 years to 20.9% during the last 3 years. Oxytocin augmentation was used in 255 (14.5%) cases, first increasing from 11.1% during the first 3 years to 18.7% in 2009 and then decreasing to 14.9% during the last 3 years. Both epidurals and oxytocin were used in 163 cases (9.3%). Comparing the 404 women (23.0%) who received epidurals and/or oxytocin with those who did not have such interventions showed that maternal age and height did not differ between the groups, but women who received epidurals and/or oxytocin had higher BMI and gave birth to heavier babies.

**Table 1:** Study population characteristics (continuous variables), mean values comparing receiving and not receiving epidurals and/or oxytocin

Variable	Epidural and/or oxytocin (*n* = 404)	Neither epidural or oxytocin (*n* = 1349)	*p*-value
	Mean (SD)	Mean (SD)	
Maternal age [years]	30.1 (4.8)	30.3 (4.7)	0.481
Maternal height [cm]	166.8 (5.8)	167.1 (5.4)	0.241
Maternal body mass index [kg/m^2^]	31.5 (5.0)	30.6 (4.6)	0.002
Gestational age [days]	280.0 (7.2)	280.9 (7.0)	0.996
Birthweight [kg]	3.88 (0.48)	3.82 (0.45)	0.022

Table 2 shows values for parity (number of previous vaginal births) and spontaneous rupture of membranes before study entry according to whether the women received epidurals and/or oxytocin infusion during the first stage of labor. Women who did not have these interventions had a lower proportion only 1 previous birth, but there was no difference in the proportion of women with spontaneous rupture of the membranes.

**Table 2:** Study population characteristics (categorical variables)

Variable	Epidural and/or oxytocin	Neither epidural or oxytocin	*p*-value
	Number (%)	Number (%)	
Parity^a^			
1	254 (62.9)	726 (53.8)	0.005
2	112 (27.7)	479 (35.5)	
≥3	37 (9.2)	143 (10.6)	
Missing	1 (0.3)	1 (0.1)	
Spontaneous rupture of membranes			
No	276 (68.3)	913 (67.7)	0.445
Yes	123 (30.5)	367 (27.2)	
Missing	5 (1.2)	69 (5.1)	

^a^Number of previous births

### Statistical modelling

Fitting a full GAMM, we found that the covariates of maternal age and maternal height did not have statistically significant effects, and these two covariates were thus excluded in the final model. There was no evidence of non-linearity in the association between BMI and conditional time (not shown), and this variable was entered as a linear predictor. There was some evidence of a non-linear relationship between birthweight and conditional time, but the effect was miniscule, and the covariate was modelled as linear to aid clinical interpretation. Although higher gestational age was associated with significantly prolonged conditional time when birthweight was removed from the model (*p* <0.001), inclusion of birthweight rendered gestational age insignificant, so gestational age was removed from the model as a predictor.

**Fig. 2:**
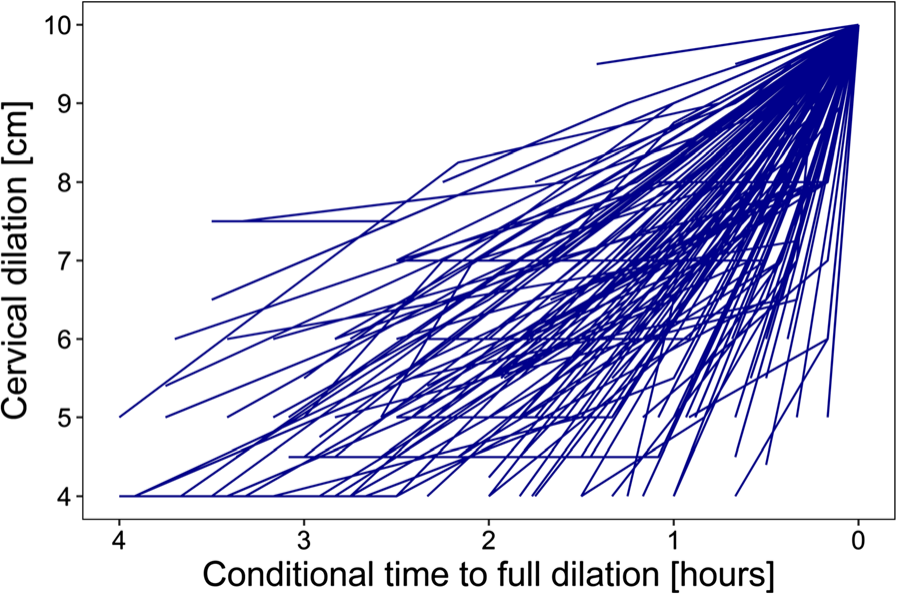
Labor curves for 10% of the study population

**Fig. 3:**
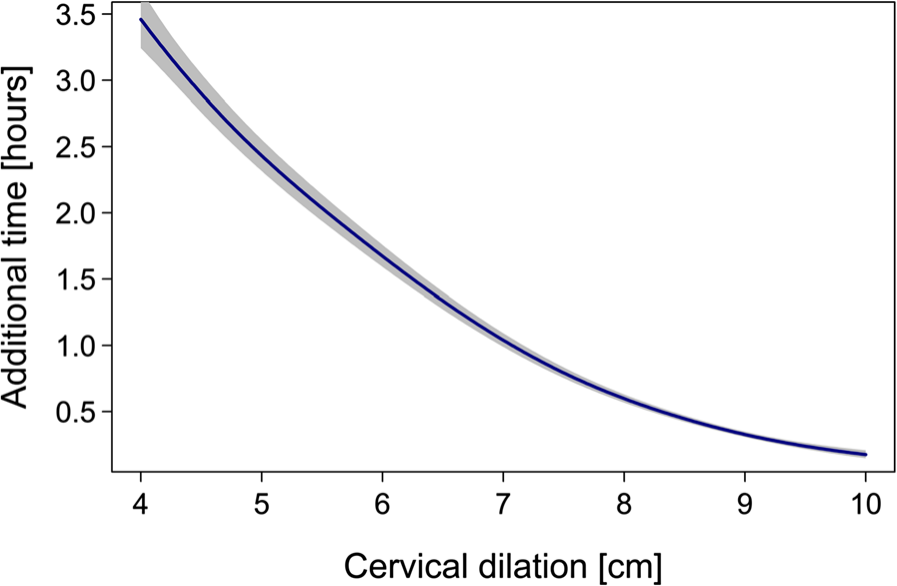
Smoothing curve for cervical dilation, depicting adjustments in the model by cervical dilation. The response value is back-transformed to the original scale (hours)

Figure 2 shows labor curves for 10% of the sample. The relationship between cervical dilation and conditional time was strongly non-linear, highlighting the need for a multiple GAMM rather than a standard linear regression model (Fig. 3).

Table 3 presents the results for the refined multiple GAMM, retaining only the significant predictors. This model explained 46% of the outcome variance (adjusted R^2^ = 0.46). Oxytocin use and birthweight were the strongest predictors. Regarding birthweight, there was a 39% increase in conditional time for every 1 kg, and oxytocin predicted 53% longer mean conditional time. Epidural use was also a strong covariate, predicting 23% longer mean conditional time, while rupture of the membranes resulted in a 31% shorter mean conditional time than for women with intact membranes.

**Table 3:** Multiple Regression Model Results Predicting Time to Completion of Cervical Dilation (Data from 1753 Partograms)

Variable	Coefficient^a^	95% CI	*p*-value
Intercept	0.449	0.320–0.629	<0.001
Body mass index [kg/m^2^]	0.990	0.983–0.998	0.010
Parity 2 vs. 1	0.905	0.840–0.976	0.009
Parity ≥3 vs. 1	0.901	0.801–1.014	0.083
Spontaneous rupture of membranes [yes]	0.763	0.707–0.824	<0.001
Epidural [yes]	1.226	1.115–1.349	<0.001
Oxytocin [yes]	1.531	1.383–1.695	<0.001
Birthweight [kg]	1.393	1.293–1.502	<0.001
Cervical dilation^b^			<0.001

^a^Values are back-transformed from log scale, resulting in a model with multiplicative effects. A coefficient of 1 thus implies no effect

^b^The coefficients for cervical dilation were estimated with a smoothing GAMM function; see Figure 3

**Fig. 4:**
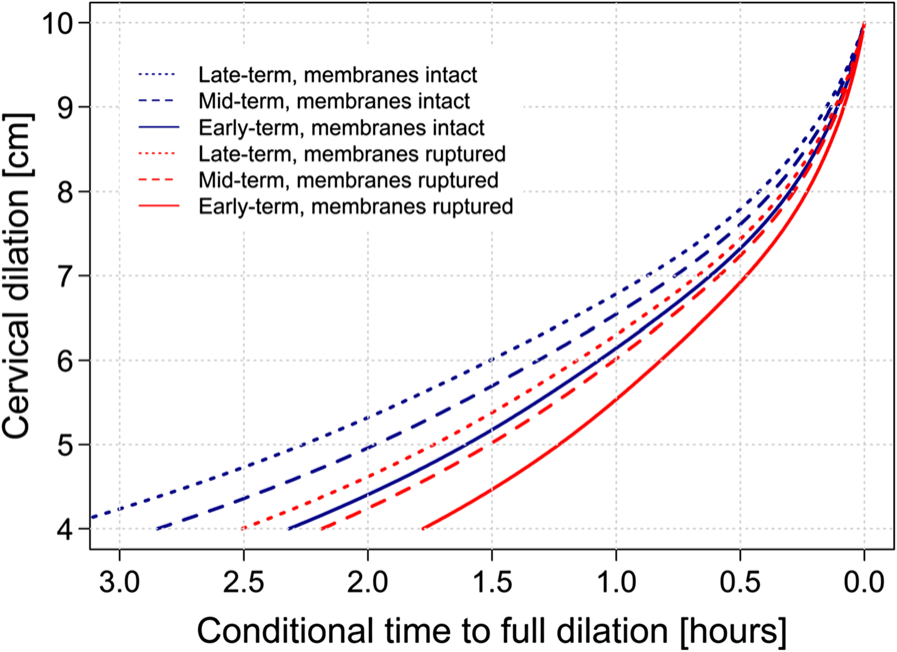
Predicted labor curves for women with membranes intact or ruptured, according gestational age. Late-term: 293 days; mid-term: 280 days; early term: 259 days

In addition to this optimal model, we also fitted another GAMM using only the covariates relevant in pre-hospital settings (age, BMI, parity, gestational length, cervical measurement, spontaneous rupture of membranes). For this, we used data only from women who did not receive oxytocin or epidural analgesia (*n* = 1349, 77%). The model coefficients are shown in the supplemental data (Additional file 1: Table S1). Using this model, we calculated predictions for conditional time for the same population and deviation from actual results. The mean absolute percentage error was 40%. Finally, we produced predictions given combinations of values for the predictors. The values for maternal age and BMI were set at an average. These results are shown in Fig. 4 (parity set at an average) and Additional file 2: Table S2.

## Discussion

Our main finding is that, in addition to cervical dilation, both birthweight and spontaneous rupture of the membranes markedly affect conditional time to completion of the first stage of labor in multiparous women. The association of fetal macrosomia (>4 kg) with longer labor and more frequent use of oxytocin has been well documented in previous research [25, 26]. In studies of labor 6 decades ago, Friedman found an large increase in the length of active nulliparous labor with increasing birthweight [27], but this association was much weaker among multipara [28]. This association was also found in the work of Nesheim [13] and in a large study which included women who delivered singleton, cephalic babies with gestational age >34 weeks at 19 hospitals in the United States [6]. This recent study analyzed birthweight in 0.5-kg increments and found that birthweight of more than 3 kg apparently became a factor in prolonging labor in multiparous women. However, a high proportion of the sample received epidurals and/or induction with oxytocin, and the researchers made no attempt to adjust for these factors. Incerti et al. found an association between gestational age and duration of active labor in nulliparous women at term but did not analyze birthweight [15]. We found evidence of collinearity between birthweight and gestational age, suggesting that the effect of gestational age is at least partially mediated by birthweight.

Artificial rupture of the membranes is performed to prevent or treat labor that progresses slowly. However, a recent review of the use of amniotomy in spontaneous labor found no evidence that it shortened the first stage of labor [20]. Unfortunately, we could not assess this association, but we found that the *spontaneous* rupture of the membranes predicted much faster progress. The clinical impression holds that spontaneous rupture often signals the descent of fetal parts and the acceleration of dilation. The apparent effect of spontaneous rupture was quite large among the subjects in the present study and could not be explained by other factors studied (e.g., different cervical measurements at admission). A likely mechanism is the reduction in uterine wall tension when the volume is reduced, as explained by LaPlace’s law [29]. This enables the uterus to maintain or generate higher pressure, which speeds up labor. Further studies on this possible explanation are warranted.

It is well known that higher parity (number of previous births) is associated with faster labor, and our findings in this regard were expected [7, 8, 13, 30, 31]. The relationship between maternal age and labor progress is unclear, although dystocia, including the need for oxytocin augmentation and prolonged labor, might increase with higher maternal age [32]. One study showed maternal age of more than 30 years to be associated with longer first stage labor (4 cm to complete dilation) in nulliparas and multiparas at term who did not receive oxytocin or epidurals [33]. However, other studies have found no association between maternal age and the duration of the first stage [13, 34]. In our previous study of unplanned out-of-institution births in Norway, we found that older women were less likely than younger women with the same parity to have unplanned out-of-institution deliveries [5]. We then speculated that this could be due to less vigorous labor or more precautions with age, and our findings from the present study support the latter.

We found that oxytocin use during the first stage of labor was associated with much longer conditional time, which is not surprising because oxytocin was exclusively used to treat slow progress. Studies have shown that early oxytocin for slow progress is associated with increased speed of labor [18, 35], and we find it likely that this was also the case in our sample. Although we have no way to offer proof, it is reasonable to assume that this relationship has more to do with the reasons or characteristics that led to oxytocin use, rather than its effects. Epidurals were also associated with longer conditional time, which can be related both to the indications for its use and to a negative effect on speed of labor [15, 17].

Maternal height has been found to be inversely related to length of labor, but we did not find this association in our sample [13]. Several studies have shown that higher maternal BMI is linked to longer labor and higher risk for caesarean delivery [36–39]. The underlying cause for this association remains poorly understood, but the effect of BMI on labor might be rather small in multiparous women [37]. We found a modest inverse relationship of a 1% reduction in the time to full dilation for an increment of 1 BMI unit.

The centralization of obstetric care which creates longer distances between homes and birthing institutions demands new solutions. The driving force behind this study was our interest in predicting time to completion of active labor and beginning of the second stage with pushing and imminent birth. The aim is not new, but the application of statistical models with complex functions might have the potential to improve prediction accuracy [15, 40]. However, some consider population-based average labor curves to be poor predictors for the individual progress of cervical dilation [30]. We tested our model in the sample and measured the mean absolute percentage error, which is a score used to measure how close predictions are to actual outcomes. Mean absolute error equally weighs all the individual differences and returns a value from 0 to infinity, with lower values indicating more accurate predictions. The predicted model outcome was not accurate enough to make clinical recommendations, but justified the generation of Fig. 4 and Additional file 2: Table S2 for exploratory purposes, clearly demonstrating the great importance of cervical measurements, gestational age, and spontaneous rupture of the membranes. For example, the estimated conditional time for a woman with 1 previous birth and 6-cm cervical dilation is 53 min with a gestational age of 259 days and spontaneously ruptured membranes but 97 min with a gestational age of 293 days and intact membranes.

Our study has many limitations. History of previous rapid labor might increase the likelihood of subsequent rapid labor, although we are not aware of any research supporting this speculation. Information about rapid or precipitous labor for our sample was not systematically recorded. We also did not include information about significant obstetric risk factors, which are thought to be uncommon in our practice. Other limitations include missing information about the artificial rupture of membranes and other factors that may be associated with the speed of labor (e.g., frequency and intensity of contractions, timing of oxytocin infusion and epidurals).

Including duration of the second stage of labor could also add value to our prediction table. However, the second stage of multiparous labor often lasts only minutes, and managing this stage en route to hospital should be avoided whenever possible. Transportation, particularly by helicopter, should also be avoided when birth is fast-approaching. In these cases, road transport by ambulance, expecting a stop en route for delivery of the baby, can be a better option.

As well, some women delivered multiple times during the study period, but we did not have permission to identify these women. This is unlikely to be a significant source of bias but is important to consider in future research. Finally, obstetric practice likely changed somewhat during the study period (e.g., increased use of epidurals), and different results may well be expected for other populations.

The strengths of this study include complete data from an entire population at a single institution meticulously collected by a small group of midwives. The population was homogeneous and consisted mostly of white women with a low prevalence of health- and social problems and strict obstetrical follow-up during pregnancy. We limited the analysis to a single Robson group of women who are too often exposed to unplanned out-of-institution births, and we excluded important confounders (e.g., previous caesarean, induction of labor and prolonged premature rupture of membranes) to avoid a “mixed bag” that clouds the issue. Labor patterns may have changed over the years, and consequently, groundbreaking studies on this important subject have lost some of their relevance [7, 28]. Thoughtful statistical modelling, considering repeated measures, adjusting for important confounders, and appropriately dealing with the complex, non-linear relationships between the predictors and outcomes enabled us to generate predictions for time to completion of cervical dilation conditional on cervical opening, parity, gestational age, birthweight, and rupture of membranes. However, due to many limitations, the study results can only be used for exploratory purposes and not for clinical recommendations. A larger prospective study could evaluate whether prediction of conditional time could be of a clinical value for parturition women in various Robson groups.

## Conclusion

Higher parity, lower birthweight (gestational age), and spontaneous rupture of the membranes are associated with shorter conditional time to completion of the first stage of labor at term among multiparous women. Multiparous women in spontaneous labor should be expected to have fast labor. Increased accuracy in the prediction of labor speed could aid clinical management, including decisions on transfers to birthing institutions.

## Additional files

Additional file 1: Table S1. Multiple Regression Model results Predicting Conditional Time Using Only Covariates Relevant in Pre-hospital Settings. (DOCX 12 kb)

Additional file 2: Table S2. Predicted Conditional Time for Parity, Gestational Length, and Cervical Dilation for Intact and Ruptured Membranes ≥4 cm. ^1^ (DOCX 14 kb)

## Abbreviations

AIC: Akaike’s information criterion;
BMI: Body mass index
CI: Confidence interval
GAM: Generalized additive models
GAMM: Generalized additive mixed models
GLM: Generalized linear models
SD: Standard deviation
