# Supplementary material for: On predicting time to completion for the first stage of spontaneous labor at term in multiparous women
Source: BMC Pregnancy Childbirth. 2017 Jun 12;17:183. doi: 10.1186/s12884-017-1345-1 (PMC5469060; doi:10.1186/s12884-017-1345-1)
Supplement: Supplementary file 1 — Multiple Regression Model results Predicting Conditional Time Using Only Covariates Relevant in Pre-hospital Settings. (DOCX 12 kb) [file 12884_2017_1345_MOESM1_ESM.docx]

**Table S1.** Multiple Regression Model results Predicting Conditional Time Using Only Covariates Relevant in Pre-hospital Settings

| **Variable** | **Coefficient**^1^ | **95% CI** |  | **p-value** |
| --- | --- | --- | --- | --- |
| Intercept | 0.099 | 0.018-0.529 | 0,529 | 0.009 |
| Maternal age [years] | 0.994 | 0.984-1.004 | 1,004 | 0.256 |
| Body mass index [kg/m^2^] | 0.993 | 0.984-1.002 | 1,002 | 0.109 |
| Parity 2 vs. 1 | 0.887 | 0.806-0.976 | 0,976 | 0.014 |
| Parity ≥3 vs. 1 | 0.885 | 0.761-1.028 | 1,028 | 0.110 |
| Spontaneous rupture of membranes [yes] | 0.769 | 0.700-0.844 | 0,844 | <0.001 |
| Gestational age [days] | 1.010 | 1.004-1.016 | 1,016 | 0.001 |
| Cervical dilation^2^ |  |  |  | <0.001 |

^1^ Values are back-transformed from log scale, resulting in a model with multiplicative effects. A coefficient of 1 thus implies no effect.

^2^ The coefficients for cervical dilation were estimated with a smoothing GAMM function.
