# Supplementary material for: On predicting time to completion for the first stage of spontaneous labor at term in multiparous women
Source: BMC Pregnancy Childbirth. 2017 Jun 12;17:183. doi: 10.1186/s12884-017-1345-1 (PMC5469060; doi:10.1186/s12884-017-1345-1)
Supplement: Supplementary file 2 — Predicted Conditional Time for Parity, Gestational Length, and Cervical Dilation for Intact and Ruptured Membranes ≥4 cm. 1 (DOCX 14 kb) [file 12884_2017_1345_MOESM2_ESM.docx]

**Table S2.** Predicted Conditional Time for Parity, Gestational Length, and Cervical Dilation for Intact and Ruptured Membranes ≥4 cm. ^1^

| Parity^2^ | Gestational length^3^ | Cervical  dilation [cm] | **Intact membranes** | | **Spontaneous rupture of membranes** | |
| --- | --- | --- | --- | --- | --- | --- |
|  |  |  | Prediction [min] | 95% CI | Prediction [min] | 95% CI |
| 1 |  | 4 | 148 | 139–158 | 114 | 104–124 |
|  | Early term | 6 | 68 | 59–78 | 53 | 43–62 |
|  |  | 8 | 19 | 10–28 | 15 | 5–24 |
|  |  | 4 | 182 | 177–188 | 140 | 133–147 |
|  | Mid-term | 6 | 87 | 83–92 | 54 | 48–60 |
|  |  | 8 | 27 | 22–31 | 8 | 2–14 |
|  |  | 4 | 209 | 202–216 | 160 | 152–169 |
|  | Late term | 6 | 97 | 90–103 | 74 | 66–82 |
|  |  | 8 | 27 | 21–33 | 21 | 13–29 |
| ≥2 |  | 4 | 128 | 118–138 | 98 | 88–108 |
|  | Early term | 6 | 59 | 50–69 | 45 | 36–55 |
|  |  | 8 | 17 | 7–26 | 13 | 3–22 |
|  |  | 4 | 158 | 152–164 | 121 | 114–128 |
|  | Mid term | 6 | 75 | 70–80 | 63 | 57–69 |
|  |  | 8 | 23 | 18–28 | 23 | 16–29 |
|  |  | 4 | 181 | 174–188 | 139 | 130–147 |
|  | Late term | 6 | 83 | 77–90 | 64 | 56–72 |
|  |  | 8 | 24 | 17–30 | 18 | 10–26 |

^1^ Spontaneous membrane rupture before first cervical examination ≥4 cm

^2^ Number of previous vaginal births

^3^ Early-term: 259 days; mid-term: 280 days; late term: 293 days
